# Genome-Wide Association Study of Periodontal Health Measured by Probing Depth in Adults Ages 18−49 years

**DOI:** 10.1534/g3.113.008755

**Published:** 2013-12-17

**Authors:** John R. Shaffer, Deborah E. Polk, Xiaojing Wang, Eleanor Feingold, Daniel E. Weeks, Myoung-Keun Lee, Karen T. Cuenco, Robert J. Weyant, Richard J. Crout, Daniel W. McNeil, Mary L. Marazita

**Affiliations:** * Department of Human Genetics, Graduate School of Public Health, University of Pittsburgh, Pittsburgh, Pennsylvania 15261; † Department of Dental Public Health and Information Management, University of Pittsburgh, School of Dental Medicine, Pittsburgh, Pennsylvania 15261; ‡ Department of Behavioral and Community Health Sciences, Graduate School of Public Health, University of Pittsburgh, Pittsburgh, Pennsylvania 15261; § Center for Craniofacial and Dental Genetics, School of Dental Medicine, University of Pittsburgh, Pittsburgh, Pennsylvania 15219; ** Department of Oral Biology, School of Dental Medicine, University of Pittsburgh, Pittsburgh, Pennsylvania 15261; †† Department of Biostatistics, Graduate School of Public Health, University of Pittsburgh, Pittsburgh, Pennsylvania 15261; ‡‡ Department of Periodontics, School of Dentistry, West Virginia University, Morgantown, West Virginia 26506; §§ Dental Practice and Rural Health, West Virginia University, Morgantown, West Virginia 26506; *** Clinical and Translational Science Institute, and Department of Psychiatry, School of Medicine, University of Pittsburgh, Pittsburgh, Pennsylvania 15213

**Keywords:** GWAS, chronic periodontitis

## Abstract

The etiology of chronic periodontitis clearly includes a heritable component. Our purpose was to perform a small exploratory genome-wide association study in adults ages 18–49 years to nominate genes associated with periodontal disease−related phenotypes for future consideration. Full-mouth periodontal pocket depth probing was performed on participants (N = 673), with affected status defined as two or more sextants with probing depths of 5.5 mm or greater. Two variations of this phenotype that differed in how missing teeth were treated were used in analysis. More than 1.2 million genetic markers across the genome were genotyped or imputed and tested for genetic association. We identified ten suggestive loci (*p*-value ≤ 1E-5), including genes/loci that have been previously implicated in chronic periodontitis: *LAMA2*, *HAS2*, *CDH2*, *ESR1*, and the genomic region on chromosome 14q21-22 between *SOS2* and *NIN*. Moreover, we nominated novel loci not previously implicated in chronic periodontitis or related pathways, including the regions 3p22 near *OSBPL10* (a lipid receptor implicated in hyperlipidemia), 4p15 near *HSP90AB2P* (a heat shock pseudogene), 11p15 near *GVINP1* (a GTPase pseudogene), 14q31 near *SEL1L* (an intracellular transporter), and 18q12 in *FHOD3* (an actin cytoskeleton regulator). Replication of these results in additional samples is needed. This is one of the first research efforts to identify genetic polymorphisms associated with chronic periodontitis-related phenotypes by the genome-wide association study approach. Though small, efforts such this are needed in order to nominate novel genes and generate new hypotheses for exploration and testing in future studies.

More than one third of the dentate adult population in the United States suffers from chronic periodontitis (Albandar *et al.* 1999), a disease in which the host response to bacteria trapped between the tooth and gum destroys both the ligament holding the tooth to the bone and the bone itself. If left untreated, periodontal disease can result in impaired mastication and possible tooth loss. A number of factors increase risk of disease, including age, smoking, and diabetes. In addition, there is evidence of a genetic component to the disease.

Two studies have examined the heritability of chronic periodontitis. In a study of 117 same-sex adult twins from the Virginia Twin Registry, the heritability of examiner-determined chronic periodontitis ranged from 48% for a measure of attachment loss to 59% for the percentage of teeth with one or more sites having attachment loss ≥2 mm (Michalowicz *et al.* 2000). In a population study of 10,578 Swedish twin pairs, the heritability of self-reported diagnosis with chronic periodontitis or presence of loose teeth was estimated to be 39% and 33% for women and men, respectively (Mucci *et al.* 2005). Taken together, the two studies provide evidence that part of the risk of disease is genetic.

On the basis of this evidence, a number of studies have attempted to identify genes that might be implicated in the disease process. Recent reviews have summarized the set of genes considered to alter risk (Zhang *et al.* 2011b, Laine *et al.* 2012). Most previous studies have adopted a candidate gene approach based on knowledge of disease pathogenesis and phenotypes. Pathogenic players for which at least some support has been obtained include pro- and anti-inflammatory mediators, the vitamin D receptor, pattern recognition receptor genes, matrix metalloproteinases, and others. The candidate gene approach is a prudent strategy for investigating hypotheses about potential disease genes for which there exists *a priori* knowledge, but unbiased, genome-wide methods are needed to nominate novel loci.

The genome-wide association study (GWAS) approach previously has been used to study periodontitis-related phenotypes. In brief, this unbiased approach scans a multitude (typically millions) of genetic variants across the entire genome one-at-a-time for statistical evidence of association. Because of the complex correlational structure of the genome (*i.e.*, linkage disequilibrium, LD), associated variants usually are not considered causal; rather, they are assumed to be proxies for unobserved causal variants. Therefore, GWAS results typically are viewed through a lens of biological plausibility, with implicated loci evaluated in terms their LD structure, the known biology of genes within an associated LD block, and of the location of these genes relative to specific associated variants. Novel candidate genes may be nominated on the basis of a combination of their physical proximity to associated variants and their compelling biological stories, although these candidate genes are not proven as disease genes via GWAS evidence alone. In this way, GWAS is extremely useful as a hypothesis-generating approach for diseases such as periodontitis, in which the genetic basis of disease is not fully understood.

Previously, the GWAS approach has been successfully used to study periodontitis-related phenotypes. A recent GWAS of the aggressive form of periodontitis has implicated *GLT6D1* (Schaefer *et al.* 2010). In a GWAS of oral bacterial profile, 13 loci demonstrated suggestive evidence of association (Divaris *et al.* 2012). In a GWAS of chronic periodontitis in 4032 German subjects (ages 20−81 years), 10 loci showed suggestive evidence of association for four related phenotypes (Teumer *et al.* 2013). Finally, in a study of 4504 European Americans ages 53−74 years, GWAS identified six suggestive loci, three for moderate chronic periodontitis, and three for severe chronic periodontitis (Divaris *et al.* 2013). However, across these studies, there has been no overlap in genes identified. Therefore, more work is needed to identify and replicate the genetic variants associated with periodontal disease-related phenotypes.

One common strategy for elucidating the role of genetics in complex outcomes is to study disease or related phenotypes in younger individuals. This approach postulates that genetic factors may play a larger role in individuals who manifest disease at a younger age and that genetic liability may be minimally obscured by cumulative effects of environmental risk factors in younger individuals. We have followed this strategy in the current work, in which we report the results of a genome-wide association scan for genetic variants affecting chronic periodontitis-related phenotypes in a comparatively young population-based cohort.

## Materials and Methods

### Participant recruitment

**Figure 1:**
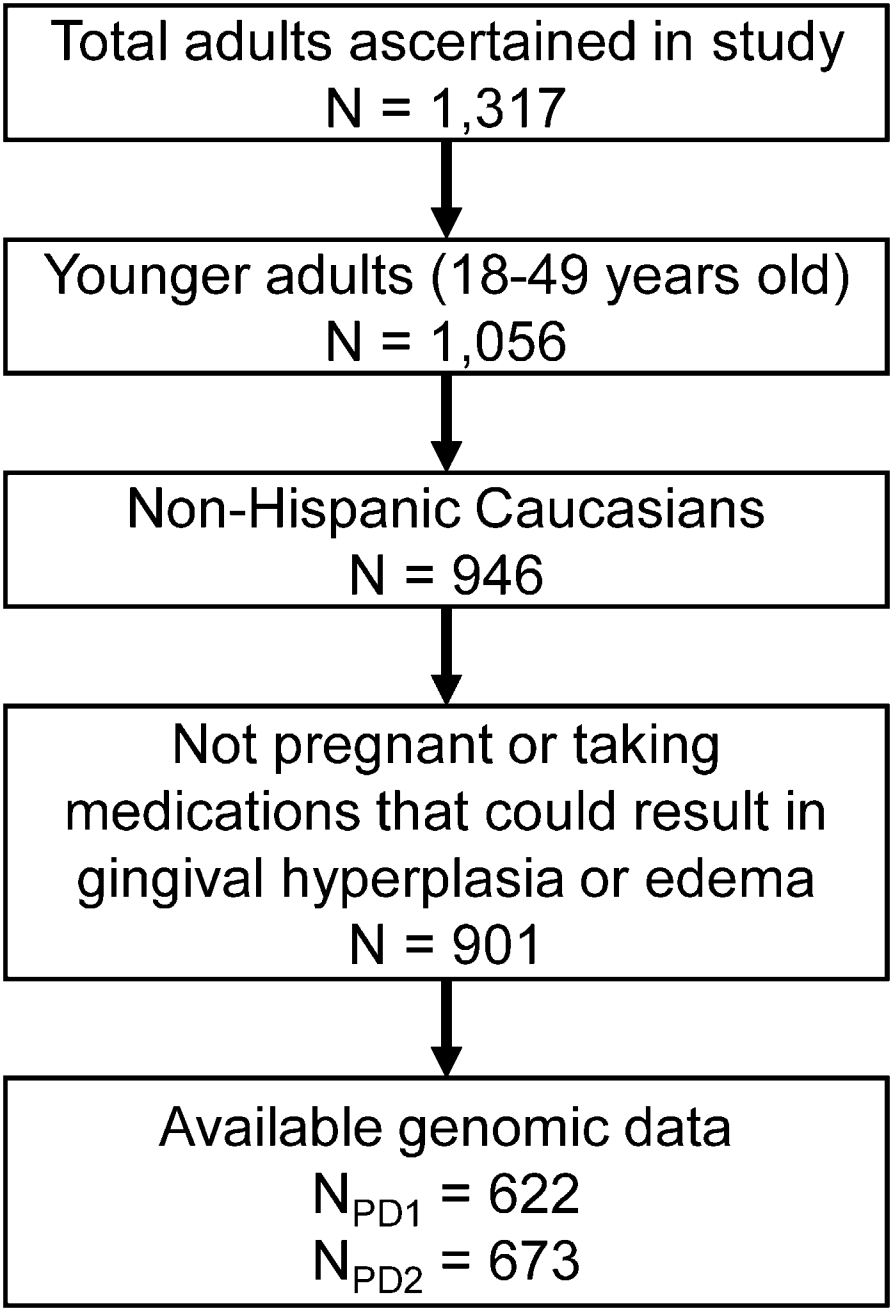
Flowchart showing numbers of participants at each stage of the study.

As previously described (Polk *et al.* 2008), the study population was ascertained through the Center for Oral Health Research in Appalachia, which recruited families from western Pennsylvania and northern West Virginia. To participate in the study, a family had to have at least one adult and at least one biologically related child between the ages of 1 and 18 who lived together. This resulted in the recruitment of 650 families. Everyone living in eligible households regardless of biological or legal relationship was invited to participate; recruitment was not based on periodontal disease or other oral health status. In the sample for this study, only adult participants (*i.e.*, in most cases the parents of a recruited family) were included, resulting in 1317 adults, of whom 1056 were between the ages of 18 and 49 (Figure 1). Written informed consent was obtained from all adult participants. All study procedures and consent forms were approved by the Institutional Review Boards of the University of Pittsburgh and West Virginia University.

### Phenotype assessment and coding

Participants received a comprehensive orodental examination by a licensed dentist or dental hygienist in a well-equipped, modern dental operatory. To assess periodontal status, every tooth was evaluated except the third molars. The research dental hygienist walked the probe around the gingival crevice. At least six areas around each tooth were examined: mesiofacial, midfacial, distofacial, and the corresponding lingual/palatal areas. To develop periodontal disease-related phenotypes, the mouth was divided into sextants, and the probing depth for the deepest pocket in each sextant was recorded as shallow (*i.e.*, <3.5 mm), moderate (*i.e.*, 3.5−5.5 mm), or deep (*i.e.*, ≥5.5 mm). If all teeth in a given sextant were missing (*i.e.*, the sextant was completely edentulous), no observation was recorded for that sextant. Two periodontal disease-related phenotypes were created that differed in how we treated missing data: (1) PD1, where we assumed the missing teeth in edentulous sextants had not been affected by chronic periodontitis; (2) PD2, where we assumed the missing teeth in edentulous sextants had been affected by chronic periodontitis. In data analysis, we coded as affected participants with at least two sextants with a pocket probing depth of at least 5.5 mm or self-reported “gum surgery” (*n* = 14) and unaffected otherwise.

### Genotyping, imputation, and quality control

Genotyping was performed at the Center of Inherited Disease Research of Johns Hopkins University as part of the GENEVA consortium using the Illumina Human610-Quadv1_B BeadChip (Illumina, San Diego, CA). Genotype imputation (inferring unobserved genotype data based on observed data) was performed using subjects from a HapMap Phase III reference panel (genetically-determined European ancestry) and resulted in approximately 1.4 million successfully imputed single-nucleotide polymorphisms (SNPs). The GENEVA consortium coordinating center at the University of Washington performed comprehensive data cleaning and quality assurance procedures (Laurie *et al.* 2010).

### Data analysis

To minimize the risk of an inflated type I error due to population stratification and to avoid a reduction in power due to genetic heterogeneity, we included in the GWAS only self-reported non-Hispanic Caucasians (*n* = 946; Figure 1). The self-reported race (ethnicity) variables showed excellent agreement with genetically determined ancestry (estimated by principal components analysis of genome-wide SNP data). To avoid other confounding factors, we excluded from our analyses those who were pregnant (*n* = 13) or who reported taking medications that could result in gingival hyperplasia or edema, including birth control pills (*n* = 24), estrogen-replacement therapy (*n* = 3), calcium channel blockers (*n* = 1), or phenytoin (*n* = 4). Because of the rural community-based recruitment strategy, our sample contained a minority of known and cryptic biological relatives, which could theoretically lead to genomic inflation (*i.e.*, *p*-values biased away from the null hypothesis) in standard association tests. Because most samples in this study (>91%) were unrelated, we did not explicitly model the relatedness among individuals; instead, we guarded against any potential bias due to population structure (*i.e.*, relatedness among participants) by closely monitoring genomic inflation factor. No bias was detected.

To test for genetic association between disease status and SNPs markers, we performed GWAS in PLINK (http://pngu.mgh.harvard.edu/~purcell/plink; Purcell *et al.* 2007) under the logistic regression model (–logistic option) while adjusting for age. The analyses were performed with both genotyped and imputed SNP data. Before analysis, HWE (*p*-value ≤ 1E-4) and minor allele frequency (MAF ≤ 0.02) filters were applied to exclude outlier or rare SNPs. We used variance components methods that condition on the known biological relationships (Almasy and Blangero 1998) in the sample to verify statistical significance of top hits. We explored all signals with “suggestive significance” (*P*-value ≤ 1E-5) using several statistical and bioinformatics tools including WGAViewer (http://compute1.lsrc.duke.edu/softwares/WGAViewer/; Ge *et al.* 2008) to search for genes near SNPs with suggestive significance, R statistical package (R Foundation for Statistical Computing, Vienna, Austria) to calculate genomic inflation factors (lambda) and generate Manhattan and quantile-quantile (Q-Q) plots, and LocusZoom (http://csg.sph.umich.edu/locuszoom/) to visualize regions around SNPs with suggestive significance. Likewise, we scrutinized SNPs in 31 previously implicated candidate genes—*IL1B*, *IL1RN*, *IL6*, *IL10*, *VDR*, *CD14*, *TLR4*, *MMP1* (Laine *et al.* 2012), *GLT6D1* (Divaris *et al.* 2012), *NPY*, *WNT5A*, *NCR2*, *EMR1* (Divaris *et al.* 2013), *EPHA3*, *RAB6C*, *C9orf150*, *IQSEC1*, *ERC2*, *CAMK4*, *MFSD1*, *LBP*, *ETS2*, *FAM180A* (Teumer *et al.* 2013), *KCNK1*, *FBXO38*, *UHRF2*, *IL33*, *RUNX2*, *TRPS1*, *CAMTA1*, *VAMP3* (Divaris *et al.* 2012)—for any evidence of association. To exclude the possibility of spurious signals caused by poor genotype calling, we also generated and visually inspected allele intensity plots for top associated SNPs.

## Results

After filtering out the non-Caucasian individuals and those whose age was beyond our focus, we found that the sample size was 901 (62.2% female), with a mean age of 32.9 years (SD = 7.7, min = 18 years, max = 48.87 years). After taking into account genotyping availability, the sample size for PD1 was 622, with 93 participants being classified as affected (15.0%), whereas the sample size for PD2 was 673, with 176 participants being classified as affected (26.2%; Figure 1). The two approaches for classifying disease resulted in different classifications for 83 participants (12.3% of the total sample), 78 of whom were completely edentulous. Both PD1 (odds ratio [OR] 1.36, 95% confidence interval [95% CI] 1.08–1.72) and PD2 (OR 1.64, 95% CI 1.35–1.99) were significantly associated with greater age.

**Figure 2:**
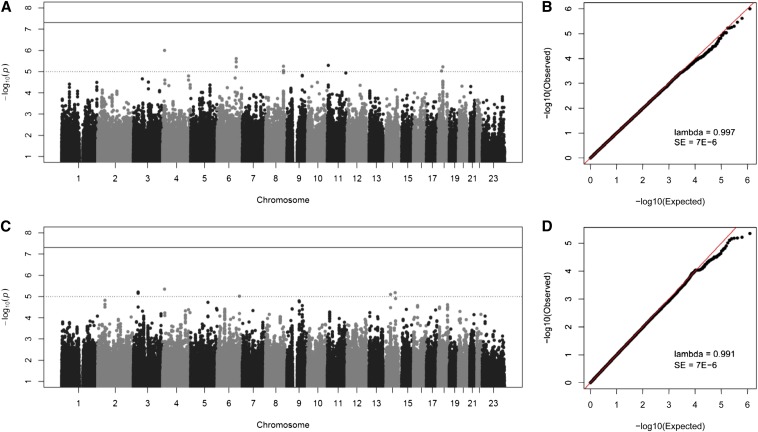
Manhattan and Q-Q plots for periodontal disease related phenotypes. (A) Manhattan plot for PD1. (B) Q-Q plot for PD1. (C) Manhattan plot for PD2. (D) Q-Q plot for PD2. All *P*-values are negative log_10_ transformed. Both genotyped and imputed SNPs are shown.

**Figure 3:**
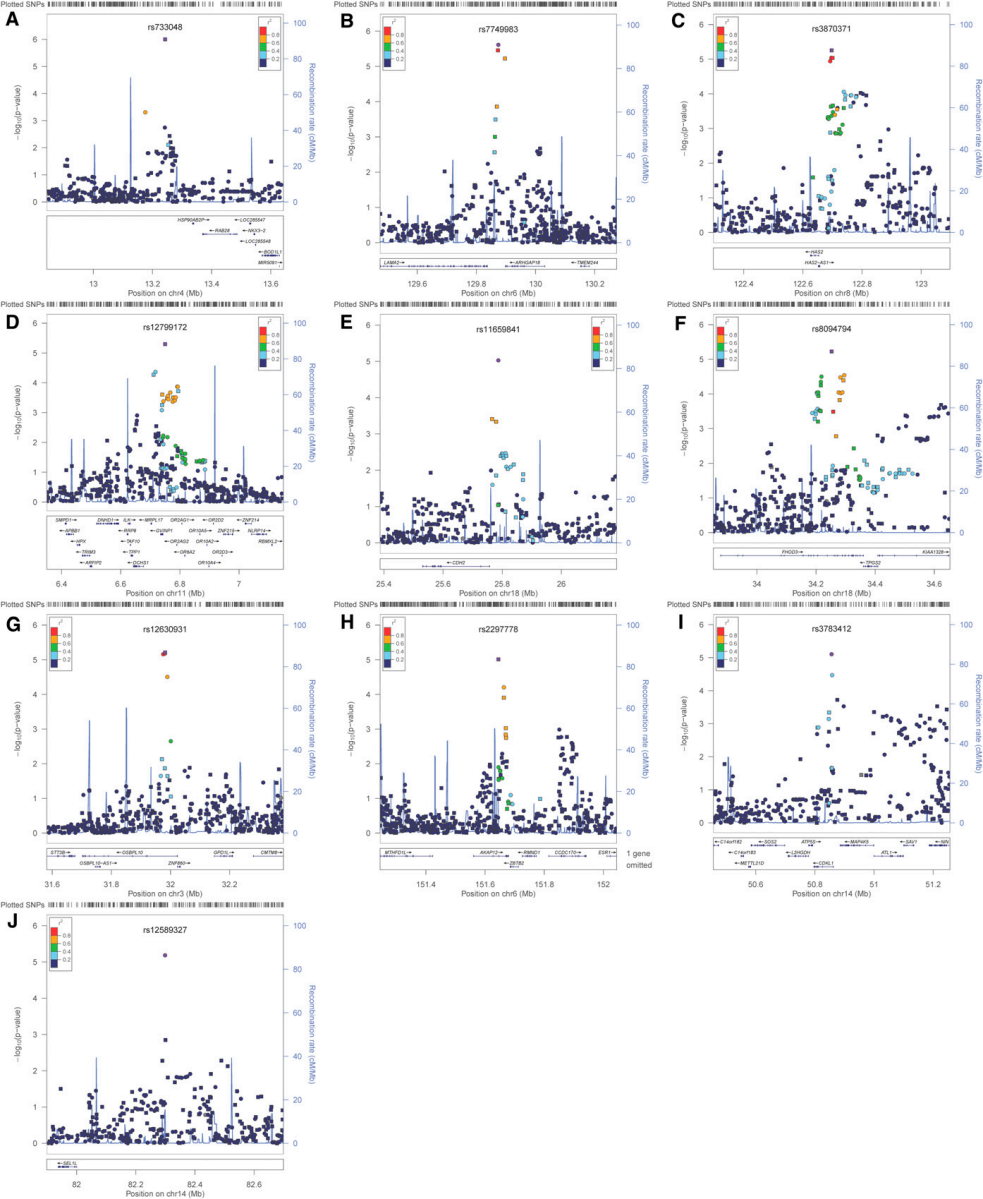
Negative log_10_ transformed *P*-values and physical positions for SNPs in associated regions. Suggestive associations for PD1: (A) SNPs on chromosome 4 occurring near the H*SP90AB2P*, *RAB28*, *BOD1L*, and *NKX3-2* genes. (B) SNPs on chromosome 6 occurring near the *LAMA2* and *ARHGAP18* genes. (C) SNPs on chromosome 8 occurring near the *HAS2* and *HAS2AS* genes. (D) SNPs on chromosome 11p15. (E) SNPs on chromosome 18 occurring near the *CDH2* gene. (F) SNPs on chromosome 18 near the *FHOD3*, *TPGS2*, and *KIAA1328* genes. Suggestive associations for PD2: (G) SNPs on chromosome 3 near the *OSBPL10*, *ZNF860*, *GPD1L*, *CMTM8*, and *STT3B* genes. (H) SNPs on chromosome 6 near the *MTHFD1L*, *AKAP12*, *ZBTB2*, *RMND1*, and *ESR1* genes. (I) SNPs on chromosome 14 near the *SOS2*, *L2HGDH*, *ATP5S*, *CDKL1*, *MAP4K5*, *ATL1*, *SAV1*, and *NIN* genes. (J) SNPs on chromosome 14 near the *SEL1L* gene. Colors indicate LD between the index SNP (purple) and other SNPs based on HapMap CEU data. The rug plot indicates regional SNP density. The recombination rate overlay (blue line, right x-axis) is based on HapMap CEU data. Gene positions and directions of transcription are annotated based on hg19/1000 Genomes Nov 2010 release.

As shown in the Manhattan plots from the GWAS (Figure 2), we did not observe any associations meeting the genome-wide level of significance (*i.e.*, *P*-value < 5E-8), which is not surprising because this threshold is incredibly conservative and our sample size is modest (in the context of GWAS). The genomic inflation factor, lambda, was 0.997 and 0.991 for PD1 and PD2, respectively, indicating that there was no inflation of *P*-values as the result of population structure or relatedness. Although no SNP passed the threshold for genome-wide significance, 10 suggestive loci, represented by 17 SNPs (10 genotyped and 7 imputed) with *P*-values between 1E-5 and 1E-7, were observed (Table 1 and Figure 3).

**Table 1:** Suggestive associations observed for periodontal disease-related phenotypes

Phenotype	Chr.	SNP	BP	MAF	OR	*P*-Value	Gene(s) within 400 kb	Corroborating Evidence
PD1	4p15	rs733048	12851895	0.22	2.40	1.0E-6	*HSP90AB2P*, *RAB28*, *BOD1L*, and *NKX3-2*	None
	6q22-23	rs10457525	129914659	0.80	2.33	3.5E-6	*LAMA2* and *ARHGAP18*	*LAMA2* is expressed in periodontal ligament and gingival fibroblasts (Han and Amar 2002) and is differentially expressed between chronically inflamed and healthy periodontal ligaments (Gersdorff *et al.* 2008)
		rs7749983	129916048	0.19	2.39	2.4E-6		
		rs10457526	129938194	0.78	2.26	6.0E-6		
	8q24	rs7816221	122765324	0.32	2.12	9.2E-6	*HAS2* and *HAS2AS*	*HAS2* and *HAS2AS* regulate hyaluronan, which is produced during tissue repair and has been shown to promote adhesion and proliferation of periodontal ligament cells (Takeda *et al.* 2011), and inhibits periodontal pathogens (Rodrigues *et al.* 2010)
		rs3870371	122766313	0.32	2.15	5.6E-6		
		rs920455	122769378	0.68	2.11	9.2E-6		
	11p15	rs12799172	6706633	0.64	2.12	5.1E-6	Many genes	none
	18q11	rs11659841	24040878	0.13	2.48	9.4E-6	*CDH2*	*CDH2*, a mediator of cell adhesion, is involved in periodontal ligament cell differentiation (Lin *et al.* 1999)
	18q12	rs8094794	32506747	0.79	2.17	5.9E-6	*FHOD3*, *TPGS2*, and *KIAA1328*	None
PD2	3p22	rs11713199	31950325	0.70	1.87	6.9E-6	*OSBPL10*, *ZNF860*, *GPD1L*, *CMTM8*, and *STT3B*	None
		rs12630254	31956231	0.30	1.90	6.7E-6		
		rs12630931	31956771	0.30	1.89	6.2E-6		
	4p15	rs733048	12851895	0.22	1.95	4.4E-6	HSP90AB2P, RAB28, BOD1L, and NKX3-2	None
	6q25	rs2297778	151686771	0.78	2.32	9.7E-6	*MTHFD1L*, *AKAP12*, *ZBTB2*, *RMND1*, and *ESR1*	*ESR1* is involved in regulating alveolar bone formation and remodeling (Zhang *et al.* 2011a) and differentiation of periodontal ligament stem cells (Pan *et al.* 2011)
	14q21	rs3783412	49926391	0.53	1.85	7.9E-6	SOS2, L2HGDH, ATP5S, CDKL1, MAP4K5, ATL1, SAV1, and NIN	This locus was the most significant association observed in an independent GWAS of severe chronic periodontitis in older adults (Divaris *et al.* 2013)
	14q31	rs12589327	81368926	0.39	2.13	6.6E-6	*SEL1L*	None

Chr, chromosome; SNP, single-nucleotide polymorphism; BP, base pair; MAF, minimum allele frequency; OR, odds ratio.

Ten SNPs across six loci exhibited suggestive evidence of association with PD1. The top-ranking SNP was rs733048 in an intergenic region on chromosome 4p15 near *HSP90AB2P*, *RAB28*, *BOD1L*, and *NKX3-2* genes. Other suggestive loci were observed on chromosome 6q22-23 near *LAMA2* and *ARHGAP18* genes, chromosome 8q24 near the *HAS2* and *HAS2AS* genes, and chromosome 11p15 in a LD block containing many genes, including several olfactory receptor genes. Two separate loci were observed on chromosome 18q11 and 18q12, near the *CDH2* gene, and near the *FHOD3*, *TPGS2*, and *KIAA1328* genes, respectively.

Seven SNPs (four genotyped, three imputed) across five loci exhibited suggestive evidence of association with PD2. As with PD1, the top ranking SNP was rs733048 on chromosome 4p15. Other suggestive loci included chromosome 3p22 near the *OSBPL10*, *ZNF860*, *GPD1L*, *CMTM8*, and *STT3B* genes, and chromosome 6q25 near the *MTHFD1L*, *AKAP12*, *ZBTB2*, *RMND1*, and *ESR1* genes. Two separate loci were observed on chromosome 14q21 near *SOS2*, *L2HGDH*, *ATP5S*, *CDKL1*, *MAP4K5*, *ATL1*, *SAV1*, and *NIN* genes, and on chromosome 14q31, near *SEL1L*.

Although not among our suggestive hits, we additionally inspected the strength of association for *a priori* candidate genes appearing in a recent review (*IL1B*, *IL1RN*, *IL6*, *IL10*, *VDR*, *CD14*, *TLR4*, *MMP1*; Laine *et al.* 2012) as well as hits from previous GWAS of aggressive periodontitis (*GLT6D1*; Schaefer *et al.* 2010), chronic periodontitis (*NPY*, *WNT5A*, *NCR2*, *EMR1*; Divaris *et al.* 2013), *EPHA3*, *RAB6C*, *C9orf150*, *IQSEC1*, *ERC2*, *CAMK4*, *MFSD1*, *LBP*, *ETS2*, *FAM180A*; Teumer *et al.* 2013), and periodontal bacterial colonization (*KCNK1*, *FBXO38*, *UHRF2*, *IL33*, *RUNX2*, *TRPS1*, *CAMTA1*, *VAMP3*; Divaris *et al.* 2012) in older individuals. Several SNPs among these genes demonstrated association at nominal levels of significance (*i.e.*, *P* < 0.05), which is expected due to chance alone, given the issue of multiple comparisons. However, we observed stronger associations between PD2 and SNPs in *CAMTA1* near the same exon in which association was previously reported (Divaris *et al.* 2012); two SNPs in *CAMTA1* (rs1750817 and rs1193169) showed association with *P* -values = 2E-4, and several other SNPs in this gene showed association at nominal significance (*i.e.*, *P* -values between 0.05 and 0.001). Likewise, 20 of 75 SNPs in *RUNX2* showed nominal association (*i.e.*, *P*-values between 0.05 and 0.001) with PD1, and 11 of 26 SNPs in *ETS2* showed nominal (*i.e.*, *P*-values between 0.05 and 0.001) with both PD1 and PD2. Although not meeting the burden of evidence necessary in a genome-wide context, given that *CAMTA1*, *RUNX2*, and *ETS2* were already nominated in a previous GWAS, our results may be interpreted as evidence of replication.

## Discussion

The current study is one of the first to investigate chronic periodontitis-related phenotypes by the GWAS approach. Neither our study, nor the three previously reported GWAS studies of chronic periodontitis-related phenotypes identified any genome-wide significant associations (Divaris *et al.* 2012, 2013; Teumer *et al.* 2013), which is not surprising, given that this threshold is extremely conservative, and power to detect such effects may be low given the sample sizes of these studies. Such hits could be used to nominate disease genes on the basis of statistical evidence alone. However, in the absence of such overwhelming statistical evidence, we have interpreted our suggestive associations (which also show very strong statistical evidence), in the context of the known biology of implicated genes. We identified 10 loci that were associated with our phenotypes, defined as two or more sextants with periodontal probing depths deeper than 5.5 mm. Scrutiny of many of these loci yielded corroborating evidence linking them periodontal health.

For instance, one of the suggestive loci implicated in PD1 was an LD-block containing *LAMA2* and *ARHGAP18*. *LAMA2* codes laminin alpha 2, a member of the multifunctional laminin family of proteins known to be expressed in periodontal ligament and gingival fibroblasts (Han and Amar 2002). A gene expression study comparing *in vivo* cells from chronically inflamed and healthy human periodontal ligaments showed nearly 3-fold down-regulation of *LAMA2* in inflamed cells (Gersdorff *et al.* 2008). Other laminins (*i.e.*, LAMA4, -B1, -B2, -B3, and -C3) also showed differential expression (between 2- and 10-fold). The *in vivo* differential expression observed for *LAMA2* (and other laminins) supports our finding that genetic variation in this gene may affect periodontal health.

Other suggestive loci identified in the analysis of PD1 have previously documented relationships with the periodontium, including *HAS2*, *HAS2AS*, and *CDH2*. HAS2 codes hyaluronan synthase 2, an enzyme that facilitates the transfer of hyaluronan across the cell membrane. *HAS2AS* (*HAS2* antisense mRNA) regulates *HAS2* mRNA levels and inhibits hyaluronan biosynthesis (Chao and Spicer 2005). Hyaluronan is produced during wound healing and tissue repair to provide a framework for the ingrowth of blood vessels and fibroblasts (Slevin *et al.* 2002). Consistent with this, hyaluronan speeds up the healing of intra-oral wounds (Hammad *et al.* 2011), promotes the adhesion and proliferation of periodontal ligament cells (Takeda *et al.* 2011), and significantly reduces the growth of two periodontal pathogens (Rodrigues *et al.* 2010). It also facilitates reductions in probing depth and bleeding on probing over 12 weeks following scaling and root planning (Johannsen *et al.* 2009) and inhibits plaque growth (Rodrigues *et al.* 2010). Thus, there is evidence that hyaluronan facilitates healing in the oral cavity and gingiva, supporting the biological plausibility of these genes.

*CDH2* codes cadherin-2 (*i.e.*, N-cadherin), which is involved in mediating calcium-ion-dependent cell adhesion (Takeichi 1987). *In vitro*, N-cadherin facilitates cell-to-cell interactions during periodontal ligament cell differentiation (Lin *et al.* 1999). *In vitro*, a proteinase produced by a periodontal pathogen induces cleavage of N-cadherin, and this cleavage is associated with loss of cell adhesion (Sheets *et al.* 2005). Thus, evidence supports the biological plausibility of *CDH2*.

For the PD2 phenotype, the most exciting suggestive association was on chromosome 14q21, which overlaps with a region previously implicated in a GWAS of chronic periodontitis in an older cohort. In the GWAS by Divaris *et al.*, the SNP rs12883458 in *NIN* was the top hit for the severe chronic periodontitis phenotype (Divaris *et al.* 2013). Although we observed association for a different SNP approximately 350 kb upstream of *NIN*, our hit was in the same LD block and our association signal was unusually broad, encompassing *NIN* and other genes in this region. It is unclear how *NIN*, which is important for anchoring microtubules for centrosomal function, may affect periodontitis. A more plausible gene in this region, which is in fact closer to our associated SNP, is *SOS2*. Although *SOS2* has not previously been implicated in periodontal health, disruption of its closely related homolog, *SOS1*, is responsible for hereditary gingival fibromatosis (Hart *et al.* 2002), suggesting a plausible role of *SOS* genes (Ras-specific exchange factors) in periodontal health. Additional studies are needed to determine the specific gene and causal variant driving association at this replicated periodontal risk locus.

Another suggestive locus identified in the analysis of PD2 was a genomic region, including the estrogen receptor (*i.e.*, ESR-α coded by *ESR1*), which has a previously documented relationship with the periodontium. By binding with receptors in periodontal tissue, including periodontal ligament stem cells, estrogen regulates the remodeling of alveolar bone, promotes bone formation, and inhibits bone resorption (*e.g.*, Zhang *et al.* 2011a). Estrogen induces periodontal ligament stem cells to differentiate into osteoblast-like cells via ESR-α (Pan *et al.* 2011). This differentiation can be down-regulated by a lentivirus-mediated siRNA targeting ESR-α (Zhang *et al.* 2011a). Finally, estrogen reverses the stimulatory effects of lipopolysaccharide on proinflammatory cytokine expression by human periodontal ligament cells (Shu *et al.* 2008). Thus *ESR1* may play a role in chronic periodontitis.

One SNP, rs733048, was implicated in GWAS of both PD1 and PD2. However, all other SNPs exhibiting suggestive association (*i.e.*, *P*-value < 1E-5) in only one scan were also nominally significant (with *P*-values on the order of 1E-3 to 1E-5), and had comparable effect sizes (odds ratios), in the other scan.

Aside from the *SOS2* to *NIN* region on chromosome 14, none of the other nine implicated loci overlapped with results from published GWAS of periodontitis (Schaefer *et al.* 2010; Divaris *et al.* 2012, 2013; Teumer *et al.* 2013). This may be due to the possible heterogeneity between different cohorts, especially differences in ages, and more importantly, the usage of different phenotypes among studies. In particular, aggressive periodontitis and periodontal pathogen colonization may have separate etiologies than the periodontal disease−related phenotypes of the current study. Even among the three GWAS studies of chronic periodontitis, which altogether considered eight different periodontitis-related phenotypes, there was no overlap in trait definitions. Nevertheless, modest evidence of replication was observed for previously nominated genes *CAMTA1*, *RUNX2*, and *ETS2*.

This study possesses several strengths, including high-quality genotyping and imputation data generated by Center of Inherited Disease Research and the GENEVA coordinating center and complementary phenotype definitions using different considerations for missing data. However, several limitations warrant further discussion. First, the pocket probing depth measure of affected status is a nonclinical measure of periodontal health. Because it uses pocket probing depth to estimate attachment loss rather than measuring attachment loss directly, it may underestimate disease in populations, especially older people with severe gingival recession (Landry and Jean 2002). Given that our population is younger, however, severe gingival recession is unlikely to be a problem. Thus, estimates based on the pocket probing depth measure may be a good surrogate for true disease status. In contrast, the clinical significance of genetic associations with our phenotype, given the young age of our sample, may not necessarily be generalizable to older, high-risk populations. Indeed, it is currently unknown whether the same genetic liabilities affect periodontal health throughout adulthood, although we speculate that at least some of the genetic factors that are important for chronic periodontitis in older individuals also affect periodontal health in younger individuals. Finally, our results lack genome-wide significance, that is, they do not meet the conservative threshold of significance needed to unequivocally prove association based on statistical evidence alone. This result is not surprising, given the limited power in our comparatively small sample size (for a GWAS) to detect the small effects of individual genetic variants. However, this limitation was lessened by interpreting the observed suggestive loci through a lens of biological plausibility, as statistical evidence alone was insufficient to fully implicate these loci. Targeted replication studies and functional analysis of nominated genes are highly desirable, as some of the loci identified via GWAS are likely to be false positive signals. Nevertheless we feel that the strong statistical evidence combined with plausible biology gives credence to our nomination of these genes as possible periodontitis genes. Unfortunately, there is currently a paucity of comparable or adequately large chronic periodontitis studies available for such replication studies.

In summary, using the GWAS approach, we identified 10 genetic loci associated with periodontal disease-related phenotypes at the suggestive significance level. Although approximately half of the implicated loci harbor genes with known functions potentially related to chronic periodontitis, the other loci/genes are novel. Hypothesis-generating studies, such as this, that seek to nominate novel genes as possible periodontitis-related loci, are much needed. These findings are important because they provide the foundation for the exploration of novel pathways through which chronic periodontitis may occur.

## References

[bib1] Albandar J. M. Brunelle J. A. Kingman A., 1999 Destructive periodontal disease in adults 30 years of age and older in the United States, 1988–1994. J. Periodontol. 70: 13–29 10052767 10.1902/jop.1999.70.1.13

[bib2] Almasy L. Blangero J., 1998 Multipoint quantitative-trait linkage analysis in general pedigrees. Am. J. Hum. Genet. 62: 1198–1211 9545414 10.1086/301844 PMC1377101

[bib3] Chao H. Spicer A. P., 2005 Natural antisense mRNAs to hyaluronan synthase 2 inhibit hyaluronan biosynthesis and cell proliferation. J. Biol. Chem. 280: 27513–27522 15843373 10.1074/jbc.M411544200

[bib4] Divaris K. Monda K. North K. E. Olshan A. F. Lange E. M., 2012 Genome-wide association study of periodontal pathogen colonization. J. Dent. Res. 91: 21S–28S 22699663 10.1177/0022034512447951 PMC3383103

[bib5] Divaris K. Monda K. L. North K. E. Olshan A. F. Reynolds L. M., 2013 Exploring the genetic basis of chronic periodontitis: a genome-wide association study. Hum. Mol. Genet. 22: 2312–2324 23459936 10.1093/hmg/ddt065 PMC3652417

[bib6] Ge D. Zhang K. Need A. C. Martin O. Fellay J., 2008 WGAViewer: software for genomic annotation of whole genome association studies. Genome Res. 18: 640–643 18256235 10.1101/gr.071571.107 PMC2279251

[bib7] Gersdorff N. Miro X. Roediger M. Geffers R. Toepfer T., 2008 Gene expression analysis of chronically inflamed and healthy human periodontal ligament cells in vivo. Dent. Res. J. 5: 1–11

[bib8] Hammad H. M. Hammad M. M. Abdelhadi I. N. Khalifeh M. S., 2011 Effects of topically applied agents on intra-oral wound healing in a rat model: a clinical and histomorphometric study. Int. J. Dent. Hyg. 9: 9–16 21226845 10.1111/j.1601-5037.2009.00410.x

[bib9] Han X. Amar S., 2002 Identification of genes differentially expressed in cultured human periodontal ligament fibroblasts *vs.* human gingival fibroblasts by DNA microarray analysis. J. Dent. Res. 81: 399–405 12097432 10.1177/154405910208100609

[bib10] Hart T. C. Zhang Y. Gorry M. C. Hart P. S. Cooper M., 2002 A mutation in the SOS1 gene causes hereditary gingival fibromatosis type 1. Am. J. Hum. Genet. 70: 943–954 11868160 10.1086/339689 PMC379122

[bib11] Johannsen A. Tellefsen M. Mikesjo U. Johannsen G., 2009 Local delivery of hyaluronan as an adjunct to scaling and root planing in the treatment of chronic periodontitis. J. Periodontol. 80: 1493–1497 19722800 10.1902/jop.2009.090128

[bib12] Laine M. L. Crielaard W. Loos B. G., 2012 Genetic susceptibility to periodontitis. Periodontol. 2000 58: 37–68 22133366 10.1111/j.1600-0757.2011.00415.x

[bib13] Landry R. G. Jean M., 2002 Periodontal screening and recording (PSR) index: precursors, utility, and limitations in a clinical setting. Int. Dent. J. 52: 35–40 11931220 10.1111/j.1875-595x.2002.tb00595.x

[bib14] Laurie, C. C., K. F. Doheny, D. B. Mirel, E. W. Pugh, L. J. Bierut, *et al.*, and GENEVA Investigators, 2010 Quality control and quality assurance in genotypic data for genome-wide association studies. Genet. Epidemiol. 34: 591–602. 10.1002/gepi.20516 PMC3061487 20718045

[bib15] Lin W. L. Chien H. H. Cho M. I., 1999 N-cadherin expression during periodontal ligament cell differentiation in vitro. J. Periodontol. 70: 1039–1045 10505807 10.1902/jop.1999.70.9.1039

[bib16] Michalowicz B. S. Diehl S. R. Gunsolley J. C. Sparks B. S. Brooks C. N., 2000 Evidence of a substantial genetic basis for risk of adult periodontitis. J. Periodontol. 71: 1699–1707 11128917 10.1902/jop.2000.71.11.1699

[bib17] Mucci L. A. Bjorkman L. Douglass C. W. Pedersen N. L., 2005 Environmental and heritable factors in the etiology of oral diseases—a population-based study of Swedish twins. J. Dent. Res. 84: 800–805 16109987 10.1177/154405910508400904

[bib18] Pan F. Zhang R. Wang G. Ding Y., 2011 Oestrogen receptors are involved in the osteogenic differentiation of periodontal ligament stem cells. Biosci. Rep. 31: 117–124 20524935 10.1042/BSR20100029

[bib19] Polk D. E. Weyant R. J. Crout R. J. McNeil D. W. Tarter R. E., 2008 Study protocol of the Center for Oral Health Research in Appalachia (COHRA) etiology study. BMC Oral Health 8: 18 18522740 10.1186/1472-6831-8-18 PMC2443132

[bib20] Purcell S. Neale B. Todd-Brown K. Thomas L. Ferreira M. A., 2007 PLINK: a tool set for whole-genome association and population-based linkage analyses. Am. J. Hum. Genet. 81: 559–575 17701901 10.1086/519795 PMC1950838

[bib21] Rodrigues S. V. Acharya A. B. Bhadbhade S. Thakur S. L., 2010 Hyaluronan-containing mouthwash as an adjunctive plaque-control agent. Oral Health Prev. Dent. 8: 389–394 21180677

[bib22] Schaefer A. S. Richter G. M. Nothnagel M. Manke T. Dommisch H., 2010 A genome-wide association study identifies GLT6D1 as a susceptibility locus for periodontitis. Hum. Mol. Genet. 19: 553–562 19897590 10.1093/hmg/ddp508

[bib23] Sheets S. M. Potempa J. Travis J. Fletcher H. M. Casiano C. A., 2005 Gingipains from *Porphyromonas gingivalis* w38 induce cell adhesion molecule cleavage and apoptosis in endothelial cells. Infect. Immun. 73: 1543–1552 15731052 10.1128/IAI.73.3.1543-1552.2005 PMC1064927

[bib24] Shu L. Guan S. M. Fu S. M. Guo T. Cao M., 2008 Estrogen modulates cytokine expression in human periodontal ligament cells. J. Dent. Res. 87: 142–147 18218840 10.1177/154405910808700214

[bib25] Slevin M. Kumar S. Gaffney J., 2002 Angiogenic oligosaccharides of hyaluronan induce multiple signaling pathways affecting vascular endothelial cell mitogenic and wound healing responses. J. Biol. Chem. 277: 41046–41059 12194965 10.1074/jbc.M109443200

[bib26] Takeda K. Sakai N. Shiba H. Nagahara T. Fujita T., 2011 Characteristics of high-molecular-weight hyaluronic acid as a brain-derived neurotrophic factors scaffold in periodontal tissue regeneration. Tissue Eng. Part A 17: 955–967 21091323 10.1089/ten.TEA.2010.0070

[bib27] Takeichi M., 1987 Cadherins: a molecular family essential for selective cell-cell adhesion and animal morphogenesis. Trends Genet. 3: 213–217

[bib28] Teumer A. Holtfreter B. Volker U. Petersmann A. Nauck M., 2013 Genome-wide association study of chronic periodontitis in a general German population. J. Clin. Periodontol. 40: 977–985 24024966 10.1111/jcpe.12154

[bib29] Zhang B. Li Y. Zhou Q. Ding Y., 2011 a Estrogen deficiency leads to impaired osteogenic differentiation of periodontal ligament stem cells in rats. Tohoku J. Exp. Med. 223: 177–186 21372518 10.1620/tjem.223.177

[bib30] Zhang J. Sun X. Xiao L. Xie C. Zuan D., 2011 b Gene polymorphisms and periodontitis. Periodontol. 2000 56: 102–124 21501239 10.1111/j.1600-0757.2010.00371.x

